# Inhalations in Pulmonary Diseases

**Published:** 1855-05

**Authors:** 


Inhalation in Pulmonary Diseases.—Inhaling the vapor of iodine and its preparations, with or without kindred drugs, rendered volatile for the purpose, is now becoming in vogue for the relief of pulmonary diseases. The practice is only a revival of Pneumatic medicine as taught and tried two centuries ago, then as now, however, too often empirically. The indiscriminate employment of inhalations is dangerous, and may prove speedily fatal, while a cautious selection of the cases by a competent diagnostician will show that there are examples of diseased lungs in which it may be safely and beneficially employed as auxiliary to other rational treatment. Physicians should give their attention to this subject, and keep inhalation out of the hands of the quacks, some of whom are making fortunes out of it, by advertising this ancient remedy as some new thing, and then treating all cases by the *month*, for a stipend payable in advance.—*N. Y. Med Jour.*

